# Impact of Olive Oil Fatty Acids and Bioactive Compounds on Cognitive Function in Adults: A Systematic Review

**DOI:** 10.3390/foods15101791

**Published:** 2026-05-18

**Authors:** Abdallah Kanaan, Christos Papaneophytou, Eleni P. Andreou

**Affiliations:** Department of Life Sciences, School of Life and Health Sciences, University of Nicosia, Cyprus 46 Makedonitissas Avenue, 2417 Nicosia, Cyprus; aakanan18@agr.just.edu.jo (A.K.); papaneophytou.c@unic.ac.cy (C.P.)

**Keywords:** olive oil, extra virgin olive oil, monounsaturated fatty acids, bioactive compounds, polyphenols, oleic acid, cognitive function, neuroprotection

## Abstract

Background: The global increase in life expectancy has led to a higher prevalence of age-related cognitive decline, highlighting the need for effective non-pharmacological interventions. This systematic review aimed to evaluate the potential effects of olive oil, particularly its bioactive compounds and fatty acid profile, on cognitive function in adults Methods: A comprehensive literature search was conducted in PubMed, Scopus, and EBSCO in accordance with PRISMA 2020 guidelines, including peer-reviewed studies published in English between 2015 and 2025. A total of six studies met the inclusion criteria and were included in the final qualitative synthesis, comprising five randomized controlled trials and one prospective cohort study. Risk of bias was assessed using the Cochrane Risk of Bias tool (RoB 2) and the Newcastle–Ottawa Scale. Results: The findings suggest that consumption of extra virgin olive oil (EVOO), particularly high-phenolic varieties, may be associated with improvements in cognitive domains such as memory, attention, executive function, and global cognition. However, the evidence is derived from a limited number of heterogeneous studies with relatively small sample sizes. Most of the available data relate to high-phenolic EVOO and olive-derived bioactive compounds, while studies directly examining the role of fatty acid composition remain limited. Proposed mechanisms include reduced blood–brain barrier permeability, enhanced brain functional connectivity, and the neuroprotective effects of compounds such as hydroxytyrosol and oleuropein. Conclusions: While the findings suggest potential cognitive benefits of EVOO, the current evidence remains preliminary and insufficient to establish causality. Therefore, results should be interpreted with caution. Further large-scale, well-designed randomized controlled trials are required to confirm these associations and clarify the specific contributions of fatty acids and bioactive compounds.

## 1. Introduction

Cognitive performance denotes the quantifiable efficiency and effectiveness with which individuals execute higher-order mental processes, including attention, memory, reasoning, problem-solving, and executive control. It encapsulates the brain’s capacity to acquire, encode, store, and retrieve information, as well as to flexibly apply acquired knowledge in adaptive and contextually appropriate ways across diverse tasks and environments [1].

Human cognitive functioning demonstrates a pronounced developmental trajectory, characterized by a steep increase from infancy through early adulthood, followed by a progressive decline across later stages of the lifespan [2]. Several health problems may lead to cognitive dysfunction, which in turn may contribute to functional disability and, in severe cases, increased mortality [3].

In recent years, the prevalence of cognitive dysfunction has increased significantly, largely due to the rise in average life expectancy. Given the limited effectiveness of current pharmacological treatments for age-related cognitive decline, there is a growing need to develop preventive strategies, particularly those related to healthy nutrition, to reduce cognitive impairment in adults [4].

Therefore, non-pharmacological strategies, particularly dietary interventions, are increasingly recognized as important approaches for managing cognitive decline. The Mediterranean diet (MeDi) is a dietary pattern traditionally followed in countries bordering the Mediterranean Sea. It is characterized by a high intake of plant-based foods such as fruits, vegetables, legumes, nuts, and whole grains; the use of olive oil as the primary source of dietary fat; moderate consumption of fish and wine; and a low intake of red meat and processed foods. This dietary pattern is rich in monounsaturated fatty acids and bioactive compounds, particularly polyphenols, which are associated with various health benefits, including cardiovascular and cognitive protection [5]. Numerous observational studies and randomized controlled trials have demonstrated that adherence to the MeDi, including regular consumption of extra virgin olive oil may be associated with maintaining cognitive health and Alzheimer’s disease; however, current evidence is limited and not conclusive [5,6,7,8]. Additionally, adherence to the MeDi has been associated with lower overall mortality rates [9].

The Mediterranean diet primarily emphasizes the consumption of plant-based foods, the use of olive oil as the main source of fat, moderate intake of fish, and moderate wine consumption with meals, while limiting the intake of animal products [10]. Extensive research has shown that extra virgin olive oil (EVOO), a key component of this dietary pattern, exerts a wide range of beneficial health effects.

Beyond its well-established role in reducing the risk of cardiovascular diseases, type 2 diabetes, and certain cancers, emerging evidence suggests that EVOO also contributes to the maintenance of cognitive function. Its antioxidant and anti-inflammatory properties are particularly relevant in delaying age-related cognitive decline, making it a valuable dietary component for promoting long-term neurological health [11,12,13].

Olive oil is a nutrient-rich fat composed primarily of monounsaturated fatty acids, especially oleic acid, which is widely recognized for its cardioprotective effects [14]. In addition, it serves as an important dietary source of bioactive compounds. Among its different forms, EVOO is considered the highest quality due to its minimal processing and superior nutritional profile. Regular consumption of EVOO has been associated with reduced mitochondrial oxidative stress, a mechanism that may help counteract age-related cognitive decline [15,16].

EVOO also contains secoiridoids such as oleuropein aglycone, a bioactive compound that has been linked to delayed cognitive decline in older adults [17].

The relationship between olive oil composition—particularly its fatty acid profile and bioactive compounds—and cognitive performance in adults remains incompletely understood. While EVOO is rich in monounsaturated fatty acids such as oleic acid and contains bioactive compounds including polyphenols (e.g., hydroxytyrosol and oleuropein), its specific contribution to cognitive outcomes has not been fully clarified. These compounds exhibit antioxidant, anti-inflammatory, and neuroprotective properties, suggesting a potential role in maintaining cognitive health [16].

Evidence from observational and interventional studies indicates that olive oil consumption may be associated with improvements in cognitive domains such as memory, attention, and executive function. For example, findings from the PREDIMED-NAVARRA randomized controlled trial demonstrated that supplementation of the Mediterranean diet with EVOO was associated with improved cognitive performance compared with a control diet [5]. However, the overall body of evidence remains heterogeneous, with substantial variability in study populations, design, olive oil composition, dosage, intervention duration, and cognitive assessment methods. This variability limits the ability to draw consistent and generalizable conclusions.

Importantly, although cognitive performance has been widely studied, there is a lack of direct evidence investigating broader constructs such as intelligence quotient (IQ), highlighting a critical gap between theoretical assumptions and empirical findings [4,5,6,7,8].

Therefore, a systematic synthesis of the available literature is warranted to critically evaluate existing evidence, identify inconsistencies, and clarify the extent to which olive oil’s fatty acid composition and bioactive compounds influence cognitive function in adults. Such an analysis is essential to inform dietary recommendations, guide clinical practice, and support future research in nutritional neuroscience and healthy aging.

The primary objective of this systematic review is to evaluate the effects of olive oil fatty acids, particularly monounsaturated fatty acids, and its bioactive compounds on cognitive function in adults. Specifically, this review aims to assess their impact on key cognitive domains, including memory, attention, executive function, and global cognition; examine differences according to olive oil composition (e.g., phenolic content); evaluate the consistency and quality of evidence across study designs; and identify existing research gaps, including key cognitive domains such as memory, attention, and executive function, while also exploring broader constructs such as intelligence as a potential research gap.

This review focuses on adult populations (≥18 years) and aims to determine whether olive oil consumption contributes to measurable improvements in cognitive health and may serve as a potential non-pharmacological strategy for preventing or delaying age-related cognitive decline.

## 2. Materials and Methods

This systematic review was conducted in accordance with the Preferred Reporting Items for Systematic Reviews and Meta-Analyses (PRISMA) 2020 guidelines [18]. A predefined protocol was developed to identify, evaluate, and synthesize studies examining the effects of olive oil fatty acids and bioactive compounds on cognitive function in adult populations.

The review protocol was registered in the International Prospective Register of Systematic Reviews (PROSPERO; registration number CRD420251173429) and is publicly available at: https://www.crd.york.ac.uk/PROSPERO/view/CRD420251173429 (accessed on 15 November 2025). No amendments to the protocol were made after registration.

The PRISMA 2020 checklist is provided in Supplementary Table S1.

### 2.1. Search Strategy

A comprehensive literature search was conducted in PubMed, Scopus, and EBSCO. This was supplemented by manual screening of the first 200 records retrieved from Google Scholar and by backward and forward citation tracking of all included studies.

The search strategy combined Medical Subject Headings (MeSH) and free-text keywords related to olive oil, fatty acids, bioactive compounds, cognition, memory, executive function, and intelligence. The search strategy included terms related to cognitive domains, such as “memory,” “attention,” “executive function,” and “intelligence,” to ensure comprehensive coverage of higher-order cognitive constructs. The inclusion of “intelligence” in the search strategy was intended to ensure comprehensive coverage of higher-order cognitive constructs; however, no studies meeting the inclusion criteria directly assessed intelligence-related outcomes. The full search strategies for all databases, including Boolean operators and applied filters, are provided in Supplementary Material S1, while a summary is presented in Table 1.

Searches were limited to peer-reviewed articles published in English between November 2015 and November 2025.

Two reviewers (A.K. and E.A.) independently screened titles and abstracts for eligibility, followed by full-text assessment of potentially relevant studies. Any disagreements were resolved through discussion or consultation with a third reviewer (C.P).

**Figure 1:**
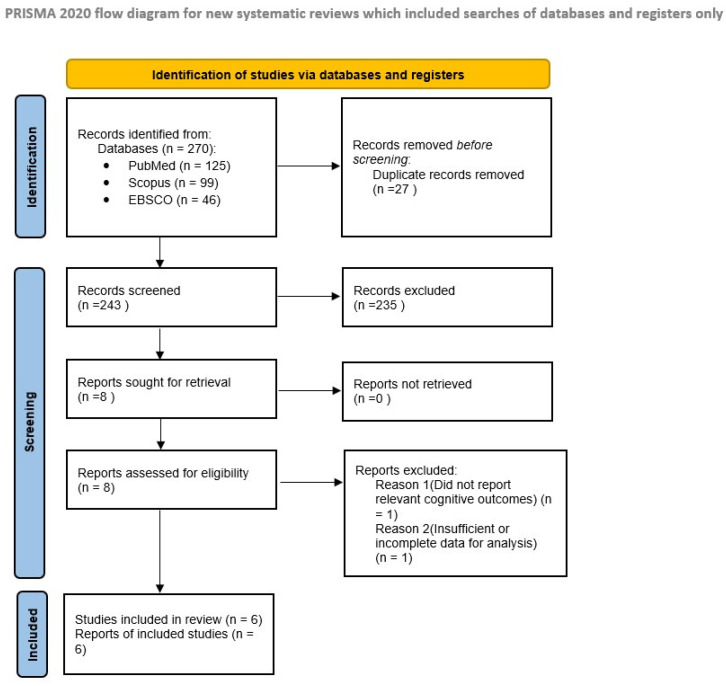
PRISMA 2020 flow diagram of the study selection process.

The study selection process was conducted in accordance with PRISMA 2020 guidelines and is presented in Figure 1.

PubMed, Scopus, and EBSCO were last searched on 15 November 2025. Google Scholar and citation tracking were conducted on the same date.

Although strict eligibility criteria were applied to ensure methodological rigor, several studies were excluded despite demonstrating partial relevance to the research question. In particular, some studies investigated olive oil consumption but did not report specific cognitive outcomes, while others assessed cognitive function without clearly defining olive oil exposure or composition. Additionally, a number of studies were excluded due to limitations in study design, such as the absence of control groups or insufficient reporting of intervention details. While these studies did not meet all inclusion criteria, their findings generally support a potential association between dietary patterns rich in olive oil and cognitive health. A brief consideration of these partially eligible studies provides additional context and highlights the need for more standardized and well-designed research in this area.

### 2.2. Eligibility Criteria

The eligibility criteria for study selection were defined according to the Population, Intervention, Comparator, Outcomes, and Study design (PICOS) framework.

Inclusion Criteria

Studies were included if they met the following criteria:

(1) Study design: Randomized controlled trials (RCTs) and observational studies (including cohort and case–control studies).
(2) Population: Human adults aged ≥18 years.
(3) Intervention/exposure: Intake or supplementation of olive oil, including extra virgin olive oil, its fatty acids (e.g., oleic acid), or bioactive compounds (e.g., polyphenols, hydroxytyrosol, oleuropein).
(4) Outcomes: Studies reporting cognitive outcomes, including memory, attention, executive function, global cognition, or other validated neuropsychological measures.
(5) Publication characteristics: Peer-reviewed articles published in English between November 2015 and November 2025.

Exclusion Criteria

Studies were excluded if they met any of the following criteria:

(1) Study design: Reviews, meta-analyses, case reports, conference abstracts, editorials, commentaries, or other non–peer-reviewed publications.
(2) Population: Non-human studies (animal or in vitro studies) or populations under 18 years of age.
(3) Intervention/exposure: Studies not assessing olive oil, its fatty acids, or bioactive compounds.
(4) Outcomes: Studies that did not report relevant cognitive or neuropsychological outcomes.
(5) Language: Articles published in languages other than English.
(6) Publication date: Studies published outside the predefined time frame (before November 2015 or after November 2025).

### 2.3. Screening Process 

All records retrieved from the electronic searches were imported into RefWorks, and duplicate records were removed. Two reviewers (A.K. and E.A.) independently screened the titles and abstracts of all identified studies according to the predefined eligibility criteria. Full texts of potentially relevant articles were then retrieved and assessed independently by the same reviewers.

Any disagreements between reviewers were resolved through discussion or consultation with a third reviewer (C.P.). No automation tools were used in the screening or selection process.

### 2.4. Data Extraction

Data extraction was conducted independently and in duplicate by two reviewers (A.K. and E.A.) using standardized data extraction forms. Extracted information included: (1) study characteristics (author, year, country, and design); (2) population characteristics and sample size; (3) intervention or exposure and comparator; (4) cognitive assessment tools and outcomes; (5) follow-up duration; and (6) main findings.

Any discrepancies between reviewers were resolved through discussion or consultation with a third reviewer (C.P.). Data extraction was performed manually without the use of automation tools, and study authors were not contacted for additional information.

The primary outcomes of interest included cognitive performance measures (e.g., ADAS-Cog, MMSE, MoCA, RAVLT, WMS-IV, reaction time, executive function) and neurobiological or imaging outcomes (e.g., blood–brain barrier permeability, functional connectivity, and Alzheimer’s disease-related biomarkers). Secondary variables included participant demographics (age, sex, education), intervention or exposure characteristics (e.g., extra virgin olive oil, phenolic content, oleic acid intake), comparators, and study-level characteristics such as design, setting, sample size, and follow-up duration. All relevant outcomes reported within each study were considered. When multiple outcome measures or time points were available, the most comprehensive and clearly reported results were extracted. Where data were missing or unclear, no imputation was performed, and only the available reported data were included in the analysis. Manufacturer and company details for commercial products and assessment tools were reported where available from the original studies.

### 2.5. Risk of Bias/Quality Assessment

Risk of bias was assessed independently by two reviewers (A.K. and E.A.) using the Cochrane Risk of Bias tool for randomized controlled trials (RoB 2) [19] and the Newcastle–Ottawa Scale (NOS) for observational studies [20]. Any disagreements between reviewers were resolved through discussion or consultation with a third reviewer (C.P.). No automation tools were used in the assessment process.

The RoB 2 tool evaluates bias across five domains: bias arising from the randomization process, deviations from intended interventions, missing outcome data, measurement of the outcome, and selection of the reported result. Each study was categorized as having low risk, some concerns, or high risk of bias.

Risk of bias assessments for randomized controlled trials are presented in Supplementary Table S5. The randomized cross-over trial was assessed separately and is reported in Supplementary Table S6.

The Newcastle–Ottawa Scale (NOS) was used to assess the quality of observational studies across three domains: selection of participants, comparability of study groups, and ascertainment of exposure or outcome. A maximum of one star was awarded per item within the selection and outcome/exposure domains, and up to two stars were assigned for comparability. Risk of bias for the cohort study is presented in Supplementary Table S7.

### 2.6. Data Synthesis

A meta-analysis was not conducted due to substantial clinical and methodological heterogeneity across studies, which precluded meaningful quantitative synthesis. The body of evidence comprised five randomized controlled trials and one observational cohort study.

Therefore, a narrative synthesis was conducted in accordance with the Synthesis Without Meta-analysis (SWiM) guidelines [21]. Studies were grouped and summarized according to study design and key cognitive outcomes, including memory, executive function, and global cognition.

The synthesis involved a structured comparison of study characteristics, intervention types, and reported outcomes to identify patterns, similarities, and differences across studies. Greater emphasis was placed on findings from randomized controlled trials due to their lower risk of bias and higher level of evidence.

The results of individual studies were summarized and presented in tabular form (Table 2), with key findings described narratively to highlight the direction and consistency of effects. Effect measures reported across studies included mean differences, changes in cognitive test scores, regression coefficients (e.g., β values), and associated *p*-values. Due to heterogeneity and the absence of meta-analysis, these measures were extracted and presented descriptively as reported in the individual studies. No formal subgroup or sensitivity analyses were conducted due to the limited number of included studies and the heterogeneity in study designs and outcome measures. However, differences in study characteristics, including study design and intervention type, were considered qualitatively during the synthesis to explore potential sources of heterogeneity.

### 2.7. Certainty of Evidence Assessment

The certainty of evidence for key outcomes was assessed independently by two reviewers using the Grading of Recommendations Assessment, Development, and Evaluation (GRADE) approach. Any disagreements were resolved through discussion or consultation with a third reviewer.

Outcomes evaluated included global cognition, memory, executive function, attention, and blood–brain barrier (BBB) integrity. Each outcome was assessed across five domains: risk of bias, inconsistency, indirectness, imprecision, and publication bias.

Based on these domains, the certainty of evidence was rated as moderate for memory and attention, low for global cognition and executive function due to heterogeneity and limited sample sizes, and very low for BBB integrity due to reliance on a single small-scale randomized controlled trial.

A detailed GRADE Summary of Findings table is provided in Supplementary Table S7.

### 2.8. Reporting Bias Assessment

Due to the small number of included studies (*n* = 6), formal assessment of reporting bias (e.g., funnel plot analysis or statistical tests) was not performed. However, potential publication bias was considered qualitatively within the GRADE framework.

## 3. Results

### 3.1. Study Selection

A total of 270 records were identified through database searching (PubMed, Scopus, and EBSCO). After removal of 27 duplicate records, 243 records were screened based on titles and abstracts, of which 235 were excluded for not meeting the inclusion criteria. Eight full-text articles were assessed for eligibility. Of these, two studies were excluded: Chang et al. (2017) [1], which did not assess olive oil or relevant exposure related to cognitive outcomes, and Lehert et al. (2015) [2], which was excluded due to ineligible study design (systematic review).

Ultimately, six studies met the inclusion criteria and were included in the final qualitative synthesis. The study selection process, including identification, screening, eligibility assessment, and inclusion, is illustrated in the PRISMA 2020 flow diagram (Figure 1).

### 3.2. Study Characteristics

A total of six studies met the eligibility criteria and were included in this systematic review, comprising five randomized controlled trials (RCTs) (Mazza et al., 2018 [13]; Yoon et al., 2023 [22]; Marianetti et al., 2022 [23]; Kaddoumi et al., 2022 [24]; Tsolaki et al., 2020 [25]) and one prospective cohort study (Sakurai et al., 2021 [26]).

The included studies were conducted in diverse geographical settings: Italy [13], Japan [22], Italy [23], USA [24], Greece [25], and one cohort study in Japan [26]. Sample sizes ranged from 18 to 154 participants. The duration of follow-up in randomized trials varied from 12 weeks to 12 months, while the cohort study assessed outcomes at a single time point.

Cognitive function was assessed using a range of validated instruments, including the Mini-Mental State Examination (MMSE), Alzheimer’s Disease Assessment Scale–Cognitive Subscale (ADAS-Cog), Montreal Cognitive Assessment (MoCA), Wechsler Memory Scale–Delayed Recall (WMS-DR), Clock Drawing Test (CDT), and Frontal Assessment Battery (FAB), among others.

A detailed summary of the characteristics of the included studies, including study design, population, interventions, comparators, and outcomes, is provided in Table 3. Additional information on geographical distribution and publication year is presented in Supplementary Tables S2 and S3.

#### 3.2.1. Randomized Clinical Trials

Detailed summary statistics and effect estimates for each included study are presented in Supplementary Table S4.

Five randomized controlled trials met the inclusion criteria, all of which used parallel design criteria. A randomized controlled trial was conducted in Greece [25] in 2020, with participants following up for 12 months at an average age of 69.8 years old. The participants were divided into three different groups: Group 1 (n = 18) was given high phenolic early harvest extra virgin olive oil, Group 2 (n = 16) was given moderate phenolic extra virgin olive oil, and Group 3 the control group (n = 16) just got the MeDi instructions. Group 1 topped and had the best performance in most cognitive measures compared to Group 2 and Group 3. Additionally, Group 2 showed significant improvement in the ADAS-Cog test (Z = −3.364, p = 0.001) and the MMSE test (Z = 2.534, p = 0.011) during the measurement period, while Group 3 demonstrated poor or similar performance to the baseline in most domains. According to the randomized controlled trial was conducted in Italy [13] in 2018, with participants followed for 12 months with an average age of 70 years. Participants were divided into two groups: the treatment group and the control group. The participants in the treatment group were given MeDi + EVOO (n = 55), and the participants in the control group were given only MeDi (n = 55). The assessment in this study was conducted using the Mini-Mental State Examination (MMSE) and the Alzheimer’s Disease Assessment Scale-Cognitive Subscale (ADAS-Cog) to evaluate any cognitive decline over a one-year period. Additionally, other validated functional and psychological tests were used, including the Verbal Fluency (VF) test and the Instrumental Activities of Daily Living (IADL) scale. Both groups experienced significant improvements in ADAS-Cog scores during the study, with an average adjusted rate decrease of −3.0 ± 0.4 from baseline. −1.6 ± 0.4 for the MeDi + EVOO and MeDi groups, respectively (p = 0.024). These results indicate the following: regular consumption of extra virgin olive oil contributes to maintaining cognitive health. No statistically significant differences were found in any other test results between the treatment group and the control group. According to the randomized controlled trial was conducted in Japan [22] in 2023. The study assessed the cognitive effects of Desert Olive Tree Pearls (DOTPs) in a Japanese community-dwelling population using a randomized, double-blind, placebo-controlled, parallel-group design. Cognitive performance was evaluated using the Cognitrax battery (BrainTrain Inc., Richmond, VA, USA). The study sample consisted of 72 middle-aged and older adults (aged between 51 and 82 years). Participants were allocated using a random method managed by an external party to ensure allocation concealment. Participants (n = 36 in each group) were assigned to receive either 3 g of DOTPs (providing approximately 32.4 mg of hydroxytyrosol daily) or a visually identical placebo made from cornstarch and soaked in olive oil, twice daily for a 12-week follow-up period. The final sample size was (n = 72), (DOTP group: 36; placebo group: 36). No significant differences in demographic and clinical variables were observed between the two groups at baseline, and the intake rate of all groups was 97.2% (97.5% and 96.8%, respectively). The primary statistically supported result is the presence of significant interaction between time and group in the domain of complex attention (p = 0.049), where the intervention group showed a significant within-group improvement (p < 0.001) compared to the placebo group (p = 0.572). While significant time effects (p < 0.05) were observed across both groups for psychomotor speed, reaction time, cognitive flexibility, processing speed, and executive function—likely attributable to practice effects or the olive oil vehicle in the placebo—the specific benefit of the high-concentration hydroxytyrosol intervention was isolated to the domain of complex attention, particularly in the older age stratum (61–82 years). According to the randomized cross-over clinical trial was conducted in Italy [23] in 2022, with participants following up for 12 months. This randomized crossover study involving 18 patients with mild Alzheimer’s disease, participants were divided into two groups: group 1 (10 patients) underwent treatment during the first six months and did not undergo treatment in the following six months, while the second group (8 patients) followed the reverse sequence. The mean age was 67 ± 5.84 years in the treatment group and 67.8 ± 4.41 years in the control group. Supplementation with oleuropein and S-acetyl glutathione achieved statistically significant improvement across almost all 14 neuropsychological measures over controls. Cognitive decline was reduced, with Mini-Mental State Examination (MMSE) scores increasing by 1.7 points (+8%) compared to no change in the control group (p = 0.0008), and Clock Drawing Test (CDT) scores improving by 0.8 points (+15%) compared to a decrease of 0.5 points (p = 0.0135). Memory gains were notable: immediate recall in the RAVLT test increased by 1.7 points (+7%) versus a decrease of 2.5 points (p = 0.0014), delayed recall rose by 0.9 points (+300%) versus a decrease of 0.5 points (p = 0.0046), and immediate recall in the RCF test improved by 2.2 points (+92%) versus a decrease of 0.75 points (p = 0.0016). Executive functions showed strong gains, including FAB (+28% vs. –15%, p < 0.0001) and PVF (+22% vs. –15%, p < 0.0001). Behavioral disturbances decreased significantly, with NPI scores decreasing by 46% versus an increase of 4% (p = 0.0001), and AES scores decreasing by 17% versus an increase of 7% (p < 0.0001). In general, the treatment led to stabilization or improvement in cognition, memory, attention, language, executive functions, and behavior despite the small sample size. According to the randomized controlled trial was conducted in USA [24] between April 2019 and February 2020, with a follow-up period of 6 months. 25 individuals completed the study. The study involved 13 participants in the EVOO group and 12 in the ROO group. Male participants represented 32% of the total participation. The study groups were well-matched, with no significant differences in average age, weight, education, MMSE, or CDR. Daily intake of EVOO has recently revealed specific neuroprotective effects when compared to ROO. Resting-state functional magnetic resonance imaging revealed increased functional connectivity between the parahippocampal gyrus and cortical areas (precentral gyri, lingual gyrus, middle frontal gyrus, superior parietal lobe) in the extra virgin olive oil group, with multiple statistically significant paired *p*-values ranging from 0.01 to 0.05, whereas refined olive oil showed no change. Blood–brain barrier (BBB) permeability was significantly reduced in participants who consumed extra virgin olive oil: left parahippocampal gyrus p = 0.00, right parahippocampal gyrus p = 0.01, left hippocampus p = 0.03, right hippocampus p = 0.04, and the values after consuming extra virgin olive oil were lower than the values after consuming refined olive oil. Refined olive oil had no effect on the blood–brain barrier’s integrity. In cognitive measures, the Clinical Dementia Rating (CDR) score improved in both groups (ROO −0.292, p = 0.0024; EVOO −0.154, p = 0.0395), with a sharp decrease in CDR-SOB scores (ROO 2.10 → 0.38, p < 0.001; EVOO 1.88 → 1.00, p < 0.001). Participants in the extra virgin olive oil group improved in total logical memory on the Wechsler scale (+2.034, p = 0.05), delayed recall (+13.451, p = 0.0377), and visual recognition (+1.462, p = 0.013), while the refined olive oil group improved in immediate visual reproduction (+3.333, p = 0.045). Biomarker analysis indicated lower plasma Aβ42/Aβ40 ratios (ROO −0.0034, p = 0.041; EVOO −0.0036, p = 0.007) and p-tau/t-tau ratios (ROO −0.347, p = 0.001; EVOO −0.286, p = 0.039), with EVOO also correlating to reduced *p*-tau181 (−0.698, p = 0.05) and increased serum NFL (+1.206 picograms/mL, p = 0.042). Overall, both oils improved clinical and behavioral outcomes, but extra virgin olive oil significantly increased brain connectivity and reduced blood–brain barrier permeability, suggesting that polyphenols are responsible for these protective benefits, while the small sample size limits generalizability.

#### 3.2.2. Prospective Cohort Study

**Figure 2:**
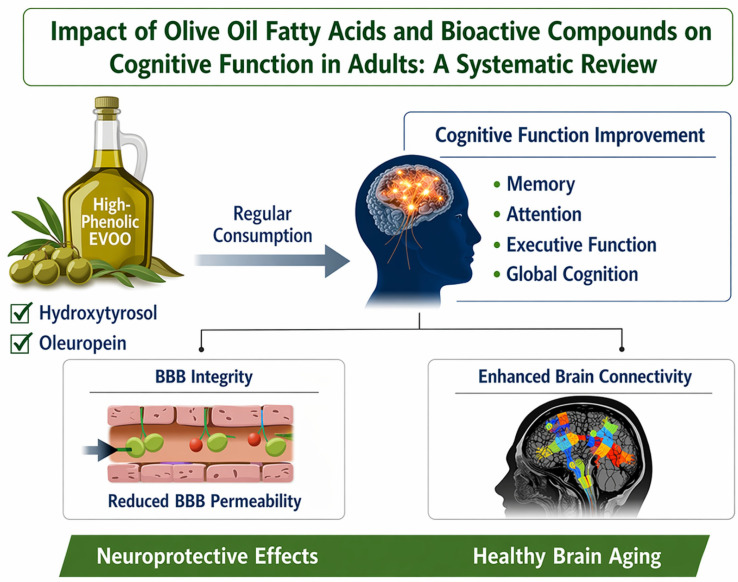
Impact of olive oil fatty acids and bioactive compounds on cognitive function in adults.

One prospective cohort study met the inclusion criteria. In a study conducted in Japan [26], recruited in 2020 and published in January 2021. In this study a group consisting of 154 community-dwelling Japanese elderly individuals (aged between 60 and 84 years; mean 73.1 ± 5.0; body mass index 22.1 ± 2.7; years of education 13.7 ± 2.2). Cognitive performance was assessed using the Montreal Cognitive Assessment (MoCA test), and episodic memory was assessed using the Wechsler Memory Scale-Delayed Recall (WMS-DR test), versus dietary consumption from the BDHQ questionnaire using stepwise multiple linear regression with age, gender, body mass index, and education as covariates. Higher daily fat intake independently predicted cognitive improvement, with statistically significant models for the MoCA scale (*R*^2^ = 0.13; *F* = 5.48; *p* = 0.0004) where fat (B = 53.6, β = 0.17, *p* = 0.0317) and age (*p* = 0.0141) were statistically significant, and for the WMS-DR scale (*R*^2^ = 0.20; *F* = 9.29; *p* < 0.0001) where fat (B = 86.1, β = 0.19, *p* = 0.0111), age (*p* = 0.0018), and education (*p* = 0.0004) were statistically significant. Intake of monounsaturated fats (MUFA) predicted higher outcomes in both the MoCA test ($R^{2}$ = 0.13; *F* = 5.66; *p* = 0.0003; MUFA B = 400, β = 0.46, *p* = 0.0157; age *p* = 0.0055) and the WMS-DR test. Oleic acid intake was a positive predictor for both the MoCA test (*R*^2^ = 0.13; *F* = 5.36; *p* = 0.0005; oleic acid B = 0.16, β = 0.16, p = 0.0405; age p = 0.0189; education p = 0.0456) and the WMS-DR test (*R*^2^ = 0.19; *F* = 9.07; *p* < 0.0001; oleic acid B = 0.25, β = 0.18, *p* = 0.0165; age *p* = 0.0026; education *p* = 0.0004). Across the models, variance inflation factors were less than 10 (no multicollinearity). Overall, the results show that higher fat intake especially monounsaturated fats and particularly oleic acid is consistently associated with better general cognitive functions and episodic memory in this group of lean elderly Japanese, with the effects remaining strong despite differences in age and education, quantitatively supported by the reported effect sizes and model fit statistics. Detailed summary statistics for each study are provided in Supplementary Table S4 and Figure 2 provides a visual summary of the effects of fatty acids and bioactive compounds in olive oil on cognitive function in adults, illustrating improvements in memory, attention, executive functions, and overall cognition, along with proposed neuroprotective mechanisms such as reducing blood–brain barrier permeability and enhancing brain connectivity.

### 3.3. Risk of Bias in Included Studies

The risk of bias across the included studies varied according to study design and methodological quality. Among the randomized controlled trials, most studies were assessed as having low risk of bias or some concerns across the evaluated domains, particularly in relation to deviations from intended interventions and measurement of outcomes. One study demonstrated a higher risk of bias due to limitations in randomization or incomplete outcome data.

The observational cohort study was assessed using the Newcastle–Ottawa Scale and was considered to be of moderate quality, with strengths in participant selection but some limitations in comparability and outcome assessment.

Detailed risk of bias assessments for individual studies are presented in Supplementary Tables S5–S7 and illustrated in Figures S1–S5.

### 3.4. Results of Synthesis

Across the included studies, olive oil consumption and its bioactive components were generally associated with improvements or preservation of cognitive function in adult populations. The most consistent findings were observed in memory and executive function domains, particularly in randomized controlled trials.

Studies investigating high-phenolic extra virgin olive oil reported significant improvements in cognitive performance measures, including ADAS-Cog, MMSE, and memory-related tests. Similarly, interventions involving hydroxytyrosol-rich compounds demonstrated benefits in specific cognitive domains such as attention and executive function.

The observational cohort study supported these findings, showing that higher intake of monounsaturated fatty acids, particularly oleic acid, was significantly associated with better cognitive performance and memory outcomes.

However, the magnitude and consistency of effects varied across studies due to differences in study design, intervention type, duration, and outcome measures. While most randomized controlled trials reported positive or stabilizing effects on cognition, some outcomes showed no statistically significant differences between intervention and control groups.

Variability in results may be explained by differences in olive oil composition (e.g., phenolic content), population characteristics, and cognitive assessment tools. No formal subgroup or sensitivity analyses were conducted; however, qualitative comparison suggested stronger effects in studies using high-phenolic olive oil and longer intervention durations.

Overall, the direction of evidence suggests a beneficial effect of olive oil and its bioactive compounds on cognitive function, although the heterogeneity and limited number of studies restrict definitive conclusions.

### 3.5. Reporting Biases

Due to the limited number of included studies (*n* = 6), formal assessment of reporting bias was not conducted. However, qualitative evaluation did not indicate clear evidence of selective reporting or publication bias. Nevertheless, the possibility of reporting bias cannot be excluded, particularly given the small sample sizes and the predominance of studies reporting positive outcomes.

### 3.6. Certainty of Evidence

The certainty of evidence for the assessed outcomes, evaluated using the GRADE approach, varied across cognitive domains. Evidence for memory and attention outcomes was rated as moderate certainty, reflecting relatively consistent findings across randomized controlled trials despite some limitations.

In contrast, evidence for global cognition and executive function was rated as low certainty due to heterogeneity in study designs, variations in interventions, and small sample sizes.

The certainty of evidence for outcomes related to blood–brain barrier integrity and neuroimaging measures was rated as very low, primarily due to reliance on a single small-scale randomized controlled trial and concerns regarding indirectness and imprecision.

Overall, the certainty of evidence across outcomes ranged from moderate to very low, highlighting the need for larger, well-designed studies to strengthen the evidence base. Detailed assessments are provided in Supplementary Table S7.

## 4. Discussion

### 4.1. Interpretation of Findings

This systematic review evaluated the effects of olive oil fatty acid composition and bioactive compounds on cognitive function in adults. Overall, the findings suggest that olive oil consumption—particularly EVOO, which is rich in monounsaturated fatty acids and polyphenols—is associated with beneficial effects on cognitive performance, including improvements in memory, attention, and executive function. However, these findings should be interpreted cautiously due to the limited number of included studies, small sample sizes, heterogeneity in study design, and the presence of studies with a high risk of bias.

The substantial heterogeneity among the included studies represents a significant challenge in interpreting the findings. The studies varied considerably in terms of participant characteristics (healthy adults, individuals with MCI, and patients with Alzheimer’s disease), intervention types (EVOO supplementation, dietary intake of oleic acid, and polyphenol-based interventions), and outcome measures. These differences limit direct comparability and may contribute to inconsistent results across studies. To address this, the findings were interpreted with consideration of population-specific and intervention-specific contexts. Overall, stronger and more consistent effects were observed in studies involving individuals with cognitive impairment compared with healthy populations, suggesting that the impact of olive oil may differ depending on baseline cognitive status.

The strongest evidence was derived from randomized controlled trials, which demonstrated that high-phenolic olive oil and hydroxytyrosol-rich interventions were associated with significant improvements in standardized cognitive measures such as ADAS-Cog, MMSE, and memory-related tests. These findings are consistent with previous research highlighting the neuroprotective properties of the Mediterranean diet and its key components, particularly olive oil, in reducing oxidative stress, inflammation, and neurodegeneration.

Observational evidence further supports these findings. The included cohort study demonstrated that higher intake of monounsaturated fatty acids, particularly oleic acid, was significantly associated with better cognitive outcomes. However, the overall body of evidence remains heterogeneous, with variations in study design, intervention protocols, olive oil composition, dosage, and outcome measures contributing to inconsistencies across studies.

This review included one cohort study and five randomized controlled trials. Despite some variability in outcomes, a generally consistent trend toward improved cognitive function was observed. Several trials indicated that EVOO, particularly high-phenolic varieties, may enhance cognitive domains such as memory, executive function, and global cognition. These effects should, however, be interpreted cautiously due to relatively small sample sizes and methodological heterogeneity.

In addition to cognitive improvements, some studies reported structural and functional brain benefits. For example, improvements in brain connectivity and blood–brain barrier integrity have been observed following EVOO consumption [24], suggesting that its effects may extend beyond symptomatic cognitive enhancement to underlying neurobiological mechanisms.

Specific studies provide further insight into these effects. Tsolaki et al. [25] reported that consumption of high-phenolic early-harvest EVOO, as well as moderate-phenolic EVOO within a Mediterranean diet, was associated with significant improvements in global cognition, letter fluency, and stabilization of mild cognitive impairment over one year. Similarly, Marianetti et al. [23] observed that a dietary supplement containing oleuropein combined with S-acetyl glutathione was associated with stabilization or improvement across multiple cognitive and behavioral measures in patients with mild Alzheimer’s disease. These findings suggest that olive polyphenols may exert synergistic neuroprotective effects through mechanisms involving the reduction in oxidative stress and amyloid toxicity.

Mazza et al. [13] also reported short-term improvements in cognitive performance among individuals following a Mediterranean diet supplemented with EVOO compared with the Mediterranean diet alone. Furthermore, Sakurai et al. [26] demonstrated that higher dietary intake of oleic acid was associated with improved general cognition and episodic memory in older adults, highlighting the importance of fatty acid composition in cognitive health.

Nevertheless, not all studies report consistent findings. Some observational studies have shown no significant association between olive oil consumption and cognitive function [27,28,29,30,31], while others have suggested potential negative associations at very high intake levels [30]. These discrepancies may be attributed to differences in study design, population characteristics, and dietary assessment methods, as well as residual confounding. High heterogeneity has also been reported in cross-sectional and cohort studies [32,33], further complicating interpretation.

From a mechanistic perspective, olive oil contains a wide range of bioactive compounds, particularly phenolic compounds such as oleuropein aglycone, which are believed to exert neuroprotective effects. These compounds have been shown to modulate oxidative stress, inflammation, and amyloid aggregation pathways, thereby contributing to the prevention of cognitive decline [13,34,35]. EVOO, produced through cold mechanical pressing without refining, preserves these bioactive components, whereas refining processes reduce their concentration and biological activity. As a result, EVOO appears to offer greater neuroprotective potential compared with refined olive oil.

The findings of this review align with a growing body of literature demonstrating the beneficial effects of the Mediterranean diet and olive oil on cognitive health. Previous systematic reviews and meta-analyses have consistently reported that adherence to the Mediterranean diet is associated with a reduced risk of Alzheimer’s disease and mild cognitive impairment [36,37,38], as well as improvements in global cognition, memory, and delayed recall [39,40,41,42]. Similarly, recent evidence indicates that EVOO consumption is associated with significant improvements in overall cognitive function in older adults [43,44]. These findings support the role of olive oil as a key component of dietary strategies aimed at promoting cognitive health and preventing neurodegenerative diseases.

Importantly, although intelligence was included as part of the search strategy to capture broader cognitive constructs, none of the included studies directly assessed IQ. This highlights a clear gap between theoretical considerations and available empirical evidence. This highlights a critical gap in the literature and underscores the need for future research to explore broader cognitive constructs beyond traditional neuropsychological measures.

The rationale for conducting this systematic review was driven by the increasing need to identify effective non-pharmacological strategies for preventing age-related cognitive decline, given the limited efficacy of current pharmacological treatments. This review provides evidence that the fatty acid profile and bioactive compounds of olive oil may play a meaningful role in supporting cognitive function in adults. However, further large-scale, well-designed randomized controlled trials are required to confirm these findings, clarify dose–response relationships, and better understand the underlying biological mechanisms.

Cognitive impairment exists along a continuum ranging from mild cognitive impairment (MCI) to more severe neurodegenerative disorders such as Alzheimer’s disease (AD). MCI is considered an intermediate stage between normal cognitive aging and dementia, characterized by measurable cognitive decline that does not yet significantly interfere with daily functioning. In contrast, Alzheimer’s disease is a progressive neurodegenerative disorder marked by the accumulation of amyloid-β plaques and hyperphosphorylated tau protein, leading to synaptic dysfunction, neuronal loss, and ultimately cognitive deterioration. Emerging evidence suggests that dietary factors may play a critical role in modulating the progression of these conditions. In particular, adherence to dietary patterns rich in extra virgin olive oil (EVOO) has been associated with slower cognitive decline and reduced risk of progression from MCI to AD. The bioactive compounds present in EVOO, including polyphenols such as hydroxytyrosol and oleuropein, may exert protective effects by reducing oxidative stress, attenuating neuroinflammation, and interfering with amyloid and tau pathology. Therefore, dietary interventions incorporating olive oil may represent a promising strategy for delaying the onset and progression of neurodegenerative disorders [40,45].

EVOO exerts neuroprotective effects through multiple molecular pathways. Its major phenolic compounds, including hydroxytyrosol and oleuropein, act as potent antioxidants by scavenging reactive oxygen species (ROS) and activating endogenous defense systems such as the Nrf2 pathway. These compounds also reduce neuroinflammation by inhibiting pro-inflammatory cytokines (e.g., TNF-α and IL-6) and modulating NF-κB signaling. Additionally, they have been shown to interfere with amyloid-β aggregation and tau phosphorylation, which are key processes in the pathogenesis of Alzheimer’s disease. Emerging evidence further suggests that EVOO polyphenols enhance synaptic plasticity, improve mitochondrial function, and preserve blood–brain barrier integrity, thereby contributing to improved neuronal resilience and cognitive performance [46].

Recent evidence further supports the role of dietary patterns and gut health in modulating cognitive function through immune-related mechanisms. For instance, Andreou and Papaneophytou (2025) [47] highlighted the critical interplay between nutrition, gut microbiota, and immune regulation, emphasizing the role of the gut–brain axis in maintaining overall health. The authors demonstrated that dietary components, particularly those characteristic of the Mediterranean diet, can influence immune responses and inflammatory processes through interactions with the gut microbiome. These mechanisms are highly relevant to cognitive health, as chronic inflammation and immune dysregulation are key contributors to neurodegenerative processes. In this context, extra virgin olive oil (EVOO), as a central component of the Mediterranean diet, may exert indirect neuroprotective effects by modulating gut microbiota composition, reducing systemic inflammation, and enhancing immune resilience. This integrative perspective supports the hypothesis that the cognitive benefits associated with olive oil consumption may extend beyond direct biochemical effects to include broader systemic interactions involving the gut–brain–immune axis.

### 4.2. Implications for Practice, Policy, and Future Research

Despite growing evidence supporting the cognitive benefits of EVOO, several important research gaps remain. Substantial heterogeneity across study designs, populations, olive oil composition (particularly phenolic content), and cognitive assessment tools limits the ability to draw definitive conclusions and hinders comparability across studies. In addition, most studies were characterized by small sample sizes and relatively short follow-up durations, reducing generalizability and limiting insight into long-term effects.

Notably, none of the included studies directly assessed broader constructs such as IQ, highlighting a disconnect between theoretical interest and empirical investigation. Furthermore, the dose–response relationship between specific bioactive compounds—such as hydroxytyrosol, oleuropein, and oleic acid—and cognitive outcomes remains unclear. There is also limited understanding of the underlying neurobiological mechanisms, including the roles of oxidative stress, neuroinflammation, and synaptic plasticity.

From a clinical and public health perspective, the findings of this review support the potential role of olive oil, particularly polyphenol-rich EVOO, as part of a dietary strategy for maintaining cognitive health and potentially delaying age-related cognitive decline. These findings are consistent with existing evidence supporting the Mediterranean diet as a protective factor against neurodegenerative diseases. However, given the variability and limited number of high-quality studies, these conclusions should be interpreted with caution.

Future research should prioritize large-scale, well-designed randomized controlled trials with standardized interventions, precise characterization of olive oil composition, and consistent, validated cognitive outcome measures. Longitudinal studies are needed to evaluate long-term effects and establish causal relationships. In addition, future investigations should incorporate biomarker and neuroimaging approaches to better elucidate underlying mechanisms, and explore broader cognitive constructs, including intelligence-related outcomes.

### 4.3. Limitations of Included Evidence

Several limitations should be considered when interpreting the findings of this review. First, the number of included studies was relatively small, which limits the generalizability of the results. In addition, substantial heterogeneity was observed across studies in terms of population characteristics, intervention types (ranging from high-phenolic olive oil to specific fatty acid supplementation), duration of exposure, and cognitive assessment tools. This variability complicates direct comparisons and reduces the overall consistency of the evidence base.

An important limitation of the current evidence base is the high risk of bias observed in several included studies. As indicated in Table 3 and Supplementary Table S6, multiple studies were rated as having a high overall risk of bias, primarily due to small sample sizes, lack of blinding, and incomplete outcome reporting. This limits the internal validity of the findings and reduces confidence in the reported cognitive benefits. Consequently, the positive effects observed in some studies should be interpreted cautiously, as they may be influenced by methodological weaknesses. Future research should prioritize rigorous study designs, including adequately powered randomized controlled trials with standardized outcome measures, to strengthen the evidence base.

Many of the included studies were characterized by small sample sizes, increasing the risk of imprecision and limiting statistical power. Furthermore, differences in olive oil composition—particularly in phenolic content—introduce additional variability that may influence cognitive outcomes. Methodological limitations were also evident in some studies, including potential bias related to randomization procedures, outcome measurement, and incomplete reporting.

The inclusion of both randomized controlled trials and a single observational cohort study introduces further complexity, particularly given differences in study design and follow-up duration. This heterogeneity limited the ability to draw definitive conclusions regarding causality and long-term effects.

Additional limitations relate to outcome and exposure assessment. The use of diverse cognitive assessment tools reduces comparability across studies, while reliance on self-reported dietary intake—especially in older adults with cognitive impairment—may introduce measurement bias.

### 4.4. Limitations of the Review Process

This review also has methodological limitations. The search was restricted to English-language publications and to studies published between 2015 and 2025, which may have introduced language and selection bias and potentially excluded relevant earlier or non-English studies.

Although a comprehensive search strategy was applied across multiple databases and supplemented by citation tracking, the possibility of publication bias cannot be entirely excluded, particularly given the small number of included studies and the tendency for positive findings to be preferentially published.

Furthermore, a meta-analysis was not conducted due to substantial heterogeneity across studies, which limited the ability to generate pooled quantitative estimates of effect. Although a structured narrative synthesis was performed in accordance with SWiM guidelines, qualitative synthesis may be more susceptible to subjective interpretation.

## 5. Conclusions

EVOO, characterized by a high content of monounsaturated fatty acids and phenolic compounds, has been increasingly investigated for its potential neuroprotective effects in adult populations. The findings from the included studies suggest that consumption of high-phenolic EVOO may be associated with improvements in several cognitive domains, including global cognition, memory, attention, and executive function. While the findings suggest potential cognitive benefits of olive oil, particularly EVOO, the current evidence remains preliminary and insufficient to establish causality.

In addition to observed cognitive outcomes, emerging evidence indicates that key bioactive components—such as oleic acid, hydroxytyrosol, and oleuropein—may contribute to neuroprotection through mechanisms involving the preservation of blood–brain barrier integrity and the enhancement of brain functional connectivity. While these findings provide biologically plausible explanations, the current evidence is not sufficient to establish definitive causal relationships.

Importantly, the overall evidence base is limited by a small number of studies, relatively small sample sizes, short intervention durations, and substantial heterogeneity in study populations, intervention types, and outcome measures. Furthermore, several included studies were identified as having a high risk of bias, which further reduces confidence in the observed effects.

Taken together, the current evidence suggests that EVOO may have a potential role as part of a non-pharmacological dietary approach to support cognitive health. Nevertheless, the available data are insufficient to draw firm conclusions regarding its effectiveness or to support specific clinical recommendations.

Future research should focus on well-designed, large-scale randomized controlled trials with standardized interventions and clearly defined olive oil compositions. Additional studies are also needed to clarify dose–response relationships, explore underlying biological mechanisms, and evaluate the effects of olive oil across different populations and stages of cognitive decline.

## Figures and Tables

**Table 1:** Search strategy used for each database.

Database	Search Terms	Filters/Notes
PubMed	(“olive oil” OR “extra virgin olive oil” OR EVOO) AND (“fatty acids” OR “oleic acid” OR “bioactive compounds” OR polyphenols OR hydroxytyrosol OR oleuropein) AND (“cognition” OR “cognitive function” OR “memory” OR “executive function” OR intelligence)	Humans; English; 2015–2025; Article types: Clinical trials, observational studies
Scopus	TITLE-ABS-KEY (“olive oil” OR “extra virgin olive oil” OR EVOO) AND TITLE-ABS-KEY (“fatty acids” OR “oleic acid” OR “bioactive compounds” OR polyphenols OR hydroxytyrosol OR oleuropein) AND TITLE-ABS-KEY (“cognition” OR “cognitive function” OR “memory” OR “executive function” OR intelligence)	English; 2015–2025; Articles; Humans
EBSCO	(“olive oil” OR “extra virgin olive oil” OR EVOO) AND (“fatty acids” OR “oleic acid” OR “bioactive compounds” OR polyphenols OR hydroxytyrosol OR oleuropein) AND (“cognition” OR “cognitive function” OR “memory” OR “executive function” OR intelligence)	Peer-reviewed; English; Adults ≥ 18 years; 2015–2025

Abbreviations: EVOO, extra virgin olive oil. Boolean operators (AND, OR) were used to combine search terms. Database-specific syntax was applied where appropriate. Filters were applied according to database functionality.

**Table 2:** Summary of included studies evaluating olive oil and cognitive function.

Study	Aim	Design & Population	Intervention	Outcomes	Key Findings	Future Directions
Mazza et al., 2018 [13]	Investigate replacing vegetable oils with EVOO in MeDi	RCT; Italians ≥ 65 (*n* = 110)	MeDi + EVOO (20–30 g/day) vs. MeDi	ADAS-Cog, MMSE	Greater improvement with EVOO (*p* = 0.024)	Larger long-term trials
Yoon et al., 2023 [22]	Effects of hydroxytyrosol-rich DOTPs	DB-RCT; adults 51–82 (*n* = 72)	3 g DOTPs vs. placebo	Cognitrax battery (BrainTrain Inc., Richmond, VA, USA)	Improved attention (*p* < 0.05)	Dose–response studies
Marianetti et al., 2022 [23]	Olive polyphenols + glutathione in AD	Cross-over RCT; mild AD (*n* = 18)	Oleuropein + glutathione	MMSE, RAVLT, FAB	Stabilization/improvement observed	Larger RCTs needed
Kaddoumi et al., 2022 [24]	EVOO vs. ROO on BBB	RCT; adults 55–75 (*n* = 25)	30 mL/day EVOO vs. ROO	MRI, MMSE	Improved BBB integrity	Long-term effects
Tsolaki et al., 2020 [25]	HP vs. MP EVOO in MCI	RCT; adults 60–80 (*n* = 50)	HP-EVOO vs. MP-EVOO vs. MeDi	ADAS-Cog, MMSE	HP-EVOO best improvement	Multicenter trials
Sakurai et al., 2021 [26]	Oleic acid intake & cognition	Cohort; adults 60–84 *(n* = 154)	Dietary intake analysis	MoCA, WMS-DR	Positive correlation with cognition	Longitudinal studies

Abbreviations: EVOO, extra virgin olive oil; MeDi, Mediterranean diet; MMSE, Mini-Mental State Examination; ADAS-Cog, Alzheimer’s Disease Assessment Scale–Cognitive; MoCA, Montreal Cognitive Assessment; RCT, randomized controlled trial; DOTPs, desert olive tree pearls; BBB, blood–brain barrier. Note: *p* < 0.05 indicates statistical significance.

**Table 3:** Baseline characteristics of included study.

Study ID/Citation	Country/Setting	Study Design	Population	Sample Size (*n*)	Intervention/Exposure	Comparator	Outcomes Measured	Follow-Up Duration	Main Findings	Risk of Bias
Mazza et al., 2018 [13]	Italy, community-based	RCT, parallel groups	Elderly ≥ 65 y, MMSE > 20	*n* = 110 (55 + 55)	MedDiet + 20–30 g/day EVOO	MedDiet alone	ADAS-cog, MMSE, ADL, IADL	12 months	EVOO group showed greater improvement in ADAS-cog vs. MedDiet alone	High
Yoon et al., 2023 [22]	Japan, community-based adults	Randomized, double-blind, RCT	Middle-aged and older adults (51–82 y)	*n* = 72 (36 DOTP, 36 placebo)	3 g DOTPs twice daily (rich in hydroxytyrosol, 162× olive oil)	Placebo in olive oil	speed, reaction time, cognitive flexibility	12 weeks	Reaction time, cognitive flexibility, processing speed, and executive function	High
Marianetti et al., 2022 [23]	Italy, Alzheimer Center	Randomized cross-over trial	Mild AD patients (IWG-2 criteria)	*n* = 18 (10 + 8)	Oleuropein + S-acetyl glutathione nutraceutical	No treatment (cross-over)	MMSE, CDT, RAVLT, FAB, NPI, AES	6 months	Significant stabilization/improvement in cognition and behavior	High
Kaddoumi et al., 2022 [24]	USA, Auburn University	RCT, blinded	Adults with Mild Cognitive Impairment	*n* = 25 (I = 13, C = 12)	30 mL/day EVOO (high polyphenols)	30 mL/day refined olive oil (ROO)	BBB permeability (MRI), fMRI connectivity, CDR, WMS-IV	6 months	EVOO reduced BBB permeability, improved connectivity and cognition	High
Tsolaki et al., 2020 [25]	Greece	Randomized, double-blind, prospective clinical trial	Older adults (60–80 y) with Mild Cognitive Impairment	*n* = 54	High Phenolic Early Harvest EVOO	Mediterranean Diet instructions only (no olive oil supplementation)	Global cognition Memory	12 months	High-phenolic EVOO improved cognition more than moderate EVOO or MeDi-only.	Low
Sakurai et al., 2021 [26] (Prospective Cohort Study)	Japan	Prospective cohort study	community-dwelling elderly ≥ 60 y	*n* = 154	fat → MUFA → oleic acid	Usual intake of other macronutrients/fatty acids	Cognitive function (MoCA, WMS-DR scores)	Single time-point assessment	Oleic acid intake significantly associated with better cognition and memory scores	Moderate

Abbreviations: AD, Alzheimer’s disease; ADAS-Cog, Alzheimer’s Disease Assessment Scale–Cognitive Subscale; AES, Apathy Evaluation Scale; BBB, blood–brain barrier; CDR, Clinical Dementia Rating; CDT, Clock Drawing Test; EVOO, extra virgin olive oil; FAB, Frontal Assessment Battery; fMRI, functional magnetic resonance imaging; IADL, Instrumental Activities of Daily Living; MeDi, Mediterranean diet; MMSE, Mini-Mental State Examination; MoCA, Montreal Cognitive Assessment; MRI, magnetic resonance imaging; MUFA, monounsaturated fatty acids; NPI, Neuropsychiatric Inventory; RAVLT, Rey Auditory Verbal Learning Test; RCT, randomized controlled trial; ROO, refined olive oil; WMS-DR, Wechsler Memory Scale–Delayed Recall.

## Data Availability

The data supporting the findings of this study are available within the article and its Supplementary Materials. Extracted data from included studies, detailed search strategies, PRISMA checklist, risk of bias assessments, and GRADE evaluations are provided in Supplementary Tables S1–S7. No analytic code was generated or used in this study. Additional materials are available from the corresponding author upon reasonable request.

## Supplementary Materials

The following supporting information can be downloaded at: https://www.mdpi.com/article/10.3390/foods15101791/s1, Table S1: PRISMA Checklist [18], Table S2: Geographical distribution of studies selected, Table S3: Distribution per year of studies selected, Table S4: Summary statistics for each study, Table S5: Risk of bias for randomized studies, Table S6: Risk of bias for randomized cross-over trial study, Table S7: Certainty of Evidence (GRADE) for Key Outcomes, Figure S1: Traffic-light risk of bias for randomized studies [13,22,24,25], Figure S2: Risk of bias for randomized studies as percentage, Figure S3: Traffic-light risk of bias for randomized cross-over trial study, Figure S4: Risk of bias for randomized cross-over trial study as percentage [23], Figure S5: Risk of bias for prospective cohort study [26], Figure S6:Traffic-light risk of bias for Prospective cohort study.

## Abbreviations

MeDi	Mediterranean diet
EVOO	Extra virgin olive oil
MUFA	Monounsaturated fatty acids
PUFA	Polyunsaturated fatty acids
ROS	Reactive oxygen species
NF-κB	Nuclear factor kappa B
Nrf2	Nuclear factor erythroid 2–related factor 2
TNF-α	Tumor necrosis factor alpha
IL-6	Interleukin 6
IQ	Intelligence quotient
RCT	Randomized controlled trial
MCI	Mild cognitive impairment
AD	Alzheimer’s disease
