# Supplementary material for: Impact of Olive Oil Fatty Acids and Bioactive Compounds on Cognitive Function in Adults: A Systematic Review
Source: Foods. 2026 May 18;15(10):1791. doi: 10.3390/foods15101791 (PMC13206388; doi:10.3390/foods15101791)
Supplement: Supplementary file 1 [file foods-15-01791-s001.zip › foods-4287336-supplementary.pdf]

Table S1. PRISMA Checklist.

Section and Topic	Item #	Checklist item	Location where Item is Reported
**TITLE**			
Title	1	Identify the report as a systematic review.	Pg.1
**ABSTRACT**			
Abstract	2	See the PRISMA 2020 for Abstracts checklist.	Pg1
**INTRODUCTION**			
Rationale	3	Describe the rationale for the review in the context of existing knowledge.	Pg 3
Objectives	4	Provide an explicit statement of the objective(s) or question(s) the review addresses.	Pg3
**METHODS**			
Eligibility criteria	5	Specify the inclusion and exclusion criteria for the review and how studies were grouped for the syntheses.	2.2
Information sources	6	Specify all databases, registers, websites, organisations, reference lists and other sources searched or consulted to identify studies. Specify the date when each source was last searched or consulted.	2.1
Search strategy	7	Present the full search strategies for all databases, registers and websites, including any filters and limits used.	2.1
Selection process	8	Specify the methods used to decide whether a study met the inclusion criteria of the review, including how many reviewers screened each record and each report retrieved, whether they worked independently, and if applicable, details of automation tools used in the process.	2.3
Data collection process	9	Specify the methods used to collect data from reports, including how many reviewers collected data from each report, whether they worked independently, any processes for obtaining or confirming data from study investigators, and if applicable, details of automation tools used in the process.	2.3
Data items	10a	List and define all outcomes for which data were sought. Specify whether all results that were compatible with each outcome domain in each study were sought (e.g. for all measures, time points, analyses), and if not, the methods used to decide which results to collect.	2.4
	10b	List and define all other variables for which data were sought (e.g. participant and intervention characteristics, funding sources). Describe any assumptions made about any missing or unclear information.	2.4
Study risk of bias assessment	11	Specify the methods used to assess risk of bias in the included studies, including details of the tool(s) used, how many reviewers assessed each study and whether they worked independently, and if applicable, details of automation tools used in the process.	2.5
Effect measures	12	Specify for each outcome the effect measure(s) (e.g. risk ratio, mean difference) used in the synthesis or presentation of results.	2.6
Synthesis	13a	Describe the processes used to decide which studies were eligible for each synthesis (e.g. tabulating the	2.6

Section and Topic	Item #	Checklist item	Location where Item is Reported
methods		study intervention characteristics and comparing against the planned groups for each synthesis (item #5)).	
	13b	Describe any methods required to prepare the data for presentation or synthesis, such as handling of missing summary statistics, or data conversions.	2.6
	13c	Describe any methods used to tabulate or visually display results of individual studies and syntheses.	2.6
	13d	Describe any methods used to synthesize results and provide a rationale for the choice(s). If meta-analysis was performed, describe the model(s), method(s) to identify the presence and extent of statistical heterogeneity, and software package(s) used.	2.6
	13e	Describe any methods used to explore possible causes of heterogeneity among study results (e.g. subgroup analysis, meta-regression).	2.6
	13f	Describe any sensitivity analyses conducted to assess robustness of the synthesized results.	2.6
Reporting bias assessment	14	Describe any methods used to assess risk of bias due to missing results in a synthesis (arising from reporting biases).	2.8
Certainty assessment	15	Describe any methods used to assess certainty (or confidence) in the body of evidence for an outcome.	2.7
**RESULTS**			
Study selection	16a	Describe the results of the search and selection process, from the number of records identified in the search to the number of studies included in the review, ideally using a flow diagram.	3.1
	16b	Cite studies that might appear to meet the inclusion criteria, but which were excluded, and explain why they were excluded.	3.1
Study characteristics	17	Cite each included study and present its characteristics.	3.2
Risk of bias in studies	18	Present assessments of risk of bias for each included study.	3.3
Results of individual studies	19	For all outcomes, present, for each study: (a) summary statistics for each group (where appropriate) and (b) an effect estimate and its precision (e.g. confidence/credible interval), ideally using structured tables or plots.	3.2.1;3.2.2
Results of syntheses	20a	For each synthesis, briefly summarise the characteristics and risk of bias among contributing studies.	3.4
	20b	Present results of all statistical syntheses conducted. If meta-analysis was done, present for each the summary estimate and its precision (e.g. confidence/credible interval) and measures of statistical heterogeneity. If comparing groups, describe the direction of the effect.	3.4
	20c	Present results of all investigations of possible causes of heterogeneity among study results.	3.4
	20d	Present results of all sensitivity analyses conducted to assess the robustness of the synthesized results.	3.4
Reporting biases	21	Present assessments of risk of bias due to missing results (arising from reporting biases) for each synthesis assessed.	3.5

Section and Topic	Item #	Checklist item	Location where Item is Reported
Certainty of evidence	22	Present assessments of certainty (or confidence) in the body of evidence for each outcome assessed.	3.6
**DISCUSSION**			
Discussion	23a	Provide a general interpretation of the results in the context of other evidence.	4.1
	23b	Discuss any limitations of the evidence included in the review.	4.3
	23c	Discuss any limitations of the review processes used.	4.4
	23d	Discuss implications of the results for practice, policy, and future research.	4.2
**OTHER INFORMATION**			
Registration and protocol	24a	Provide registration information for the review, including register name and registration number, or state that the review was not registered.	2
	24b	Indicate where the review protocol can be accessed, or state that a protocol was not prepared.	2
	24c	Describe and explain any amendments to information provided at registration or in the protocol.	NA
Support	25	Describe sources of financial or non-financial support for the review, and the role of the funders or sponsors in the review.	Pg7
Competing interests	26	Declare any competing interests of review authors.	Pg7
Availability of data, code and other materials	27	Report which of the following are publicly available and where they can be found: template data collection forms; data extracted from included studies; data used for all analyses; analytic code; any other materials used in the review.	Pg7

From: [18].

Data S1. Search Strategy

1. **PubMed:**

("Olive Oil"[MeSH Terms] OR "Fatty Acids, Monounsaturated"[MeSH Terms] OR "Phenols"[MeSH Terms] OR "Polyphenols"[MeSH Terms] OR "Bioactive Compounds" OR "Hydroxytyrosol" OR "Oleuropein") AND ("Cognition"[MeSH Terms] OR "Cognitive Function" OR "Cognitive Performance" OR "Cognition Disorders"[MeSH Terms] OR "Memory"[MeSH Terms] OR "Executive Function"[MeSH Terms] OR "Intelligence"[MeSH Terms]) AND ("Adult"[MeSH Terms] OR "Middle Aged"[MeSH Terms] OR "Aged"[MeSH Terms]) AND ("Systematic Review"[Publication Type] OR "Meta-Analysis"[Publication Type] OR "Randomized Controlled Trial"[Publication Type] OR "Clinical Trial"[Publication Type]) AND ((y_10[Filter]) AND (ffrft[Filter]) AND (medline[Filter]) AND (fha[Filter]) AND (clinicaltrial[Filter] OR duplicatepublication[Filter] OR observationalstudy[Filter] OR randomizedcontrolledtrial[Filter]) AND (humans[Filter]) AND (female[Filter] OR male[Filter]) AND (english[Filter]) AND (allchild[Filter] OR alladult[Filter] OR youngadult[Filter] OR adult[Filter] OR middleagedaged[Filter] OR middleaged[Filter] OR aged[Filter] OR 80andover[Filter]))

2. **Scopus:**

TITLE-ABS-KEY ("olive oil" OR "extra virgin olive oil" OR EVOO OR "olive oil polyphenols" OR hydroxytyrosol OR oleuropein OR "monounsaturated fatty acids" OR MUFA) AND TITLE-ABS-KEY ("cognitive performance" OR "cognitive function" OR cognition OR intelligence OR memory OR "executive function" OR "cognitive decline" OR "neuroprotection" OR "neurodegenerative diseases") AND TITLE-ABS-KEY ("adult" OR "elderly" OR "human study" OR "clinical trial" OR "randomized controlled trial" OR RCT) AND (LIMIT-TO (PUBYEAR , 2021) OR LIMIT-TO (PUBYEAR , 2022) OR LIMIT-TO (PUBYEAR , 2023) OR LIMIT-TO (PUBYEAR , 2024) OR LIMIT-TO (PUBYEAR , 2025)) AND (LIMIT-TO (SUBJAREA , "MEDI") OR LIMIT-TO (SUBJAREA , "AGRI") OR LIMIT-TO (SUBJAREA , "BIOC") OR LIMIT-TO (SUBJAREA , "NEUR")) AND (LIMIT-TO (DOCTYPE , "ar")) AND (LIMIT-TO (EXACTKEYWORD , "Human") OR LIMIT-TO (EXACTKEYWORD , "Male") OR LIMIT-TO (EXACTKEYWORD , "Humans") OR LIMIT-TO (EXACTKEYWORD , "Female")) AND (LIMIT-TO (LANGUAGE , "English")) AND (LIMIT-TO (OA , "all"))

3. **EBSCO:**

(("olive oil") AND (((((("fatty acid") OR (phenol)) OR (polyphenol)) OR ("bioactive compound")) OR (Hydroxytyrosol)) OR (Oleuropein))) AND ((((cogniti*) OR (memory)) OR ("executive function")) OR (intelligence))

Table S2. Geographical distribution of studies selected.

Country	Total
**Greece**	1
**USA**	1
**Japan**	2
**Italy**	2

Table S3. Distribution per year of studies selected.

Year	Total
**2018**	1
**2020**	1
**2021**	1
**2022**	2
**2023**	1

Table S4. Summary statistics for each study.

Study ID	Outcome Measure	Summary Statistics (Intervention vs. Control/Baseline)	Effect Estimate & Precision (*p*-value)
**Mazza et al., 2018 [13]**	Global Cognition (ADAS-Cog)	EVOO + MedDiet: -3.0 ± 0.4 decrease (improvement) vs. MedDiet alone: -1.6 ± 0.4.	Difference significant at $p=0.024$.
**Yoon et al., 2023 [22]**	Complex Attention	DOTP Group: significant within-group improvement ($p<0.001$) vs. Placebo: no significant change ($p=0.572$).	Interaction (Time x Group): $p=0.049$; specific benefit in older stratum (61–82 yrs).
**Marianetti et al., 2022 [23]**	Global Cognition (MMSE)	Treatment: +1.7 pt increase (+8%) vs. Control: 0 change.	$p=0.0008$.
	Delayed Recall (RAVLT)	Treatment: +0.9 pt increase (+300%) vs. Control: -0.5 decrease.	$p=0.0046$.
	Executive (Clock Drawing)	Treatment: +0.8 pt increase (+15%) vs. Control: -0.5 decrease.	$p=0.0135$.
	Behavior (NPI)	Treatment: -46% decrease vs. Control: +4% increase.	$p=0.0001$.
**Tsolaki et al., 2020 [25]**	Global Cognition (ADAS-Cog)	High-Phenolic EVOO showed greatest improvement compared to Moderate-Phenolic and Control.	Moderate-Phenolic vs. Control: $Z=-3.364$, $p=0.001$.

	Global Cognition (MMSE)	High-Phenolic EVOO topped performance; Control remained stable at baseline.	Moderate-Phenolic vs. Control: Z=2.534, p=0.011.
**Kaddoumi et al., 2022 [24]**	Clinical Status (CDR-SOB)	EVOO: 1.88 → 1.00 (p<0.001) vs. ROO: 2.10 → 0.38 (p<0.001).	Significant improvement in both groups (p<0.001).
	Delayed Recall (WMS)	EVOO group: Mean increase of +13.451	p=0.0377.
	BBB Permeability (L. Parahippocampal)	EVOO significantly reduced permeability; Refined Olive Oil (ROO) had no effect.	p=0.00 (values for EVOO lower than ROO).
**Sakurai et al., 2021 [26]**	Global Cognition (MoCA)	Predictor: Daily Oleic Acid Intake (R^2=0.13).	B=0.16, β=0.16, p=0.0405.
	Episodic Memory (WMS-DR)	Predictor: Daily Oleic Acid Intake (R^2 =0.19).	B=0.25, β=0.18, p=0.0165.

Table S5. Risk of bias for randomized studies.

Study ID	D1	D2	D3	D4	D5	Overall
Mazza et al., 2018 [13]	Some concerns	High Risk	High Risk	Low Risk	High Risk	High Risk
Yoon et al.,2023 [22]	Some concerns	Low Risk	High Risk	Low Risk	High Risk	High Risk
Kaddoumi et al., 2022 [24]	High Risk	Low Risk	No Information	Low Risk	High Risk	High Risk
Tsolaki et al., 2020 [25]	Low Risk	Low Risk	Low Risk	Low Risk	Low Risk	Low Risk

D1: Bias arising from the randomization process

D2: Bias due to deviations from intended interventions

D3: Bias due to missing outcome data

D4: Bias in measurement of the outcome

D5: Bias in selection of the reported result

Table S6. Risk of bias for randomized cross-over trial study.

Study ID	D1	DS	D2	D3	D4	D5	Overall
**Marianetti et al., 2022 [23]**	Some concerns	Some concerns	Some concerns	Low	High	High	High

Where:

D1: Bias arising from the randomization process

DS: Domain S: Risk of bias arising from period and carryover effects

D2: Bias due to deviations from intended interventions

D3: Bias due to missing outcome data

D4: Bias in measurement of the outcome

D5: Bias in selection of the reported result

Table S7. Certainty of Evidence (GRADE) for Key Outcomes.

Outcome	No. of Studies	Risk of Bias	Inconsistency	Indirectness	Imprecision	Publication Bias	Overall Certainty
Global cognition (ADAS-Cog, MMSE, MoCA, CDR)	5 RCTs + 1 Cohort	Some concerns	Moderate	Low	Serious	Suspected	**Low**
Memory (RAVLT, WMS-DR, CDT, Logical Memory)	4 RCTs + 1 Cohort	Some concerns	Moderate	Low	Serious	Suspected	**Low**
Executive Function / Attention (FAB, PVF, SVF)	3 RCTs	Low	Moderate	Low	Serious	Suspected	**Low**
BBB integrity / fMRI connectivity	1 RCT	Low	Not Applicable	Moderate	Serious	Suspected	**Low**
AD / MCI progression	2 RCTs	Some concerns	Moderate	Low	Serious	Suspected	**Low**

Figure S1. Traffic-light risk of bias for randomized studies [13,22,24,25].

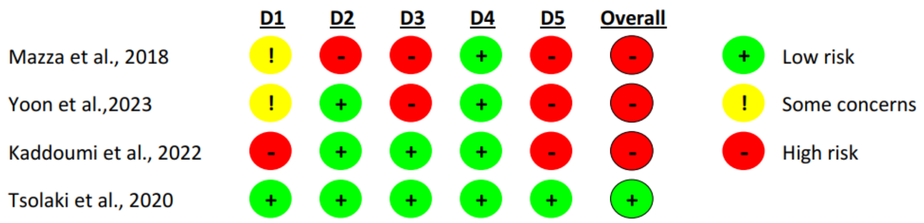

Figure S2. Risk of bias for randomized studies as percentage.

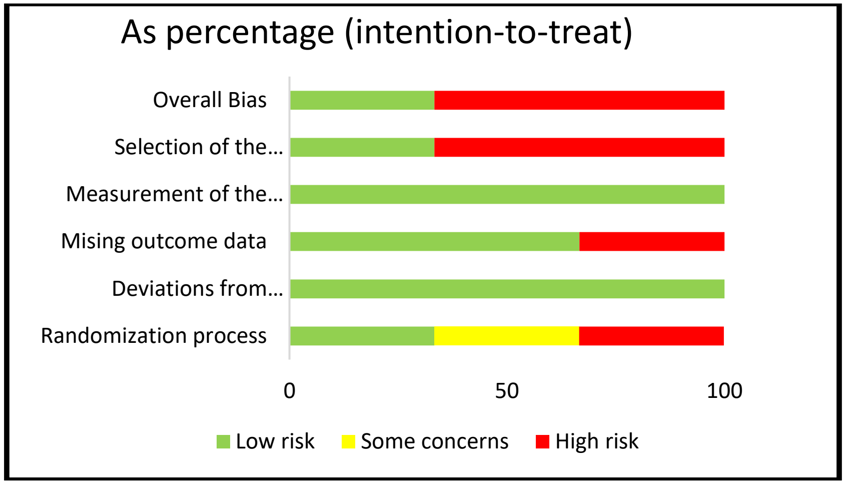

Figure S3. Traffic-light risk of bias for randomized cross-over trial study.

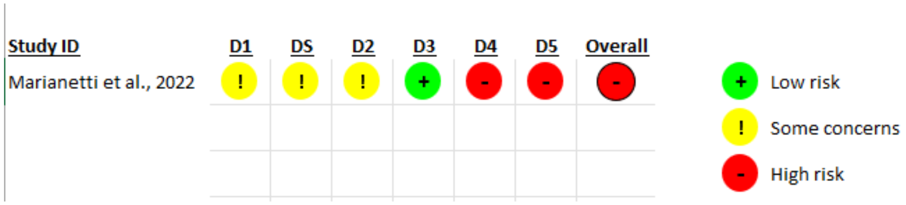

Figure S4. Risk of bias for randomized cross-over trial study as percentage [23].

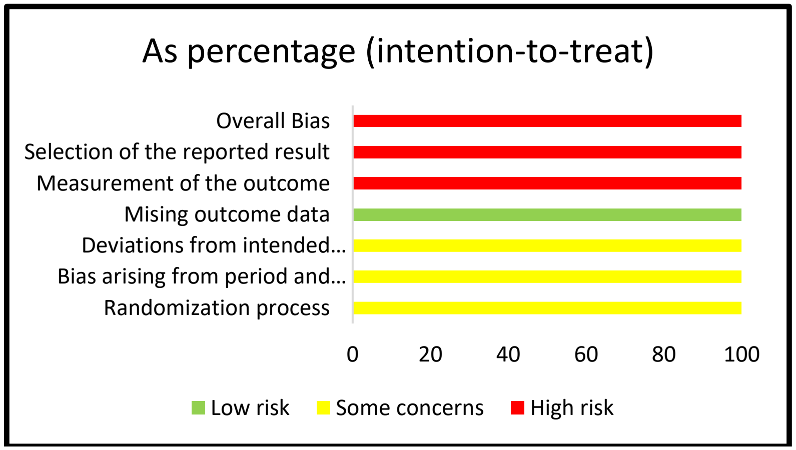

Figure S5. Risk of bias for Prospective cohort study [26].

1st author & year	Selection 4				Comparability 2	Outcome 3			FINAL SCORE AND COMMENT		Ebtesam's Notes<br>7–9 stars = Low risk<br>4–6 stars = Moderate risk<br>0–3 stars = High risk
	Representativeness of the exposed cohort	Selection of the non exposed cohort	Ascertainment of exposure	Demonstration that outcome of interest was not present at start of study	Comparability of cohorts on the basis of the design or analysis	Assessment of outcome	Was follow-up long enough for outcomes to occur	Adequacy of follow up of cohorts	Total (0–9)	Comment	
Sakurai K et al.,2021 (Prospective Cohort Study)	1	1	1	1	1	1	0	0	6	Moderate risk of bias	The group studied came from one city only, exposure was measured by self-reported diet questionnaires, there was no separate non-exposed group, without follow-up to track changes in cognition.

Figure S6. Traffic-light risk of bias for Prospective cohort study.

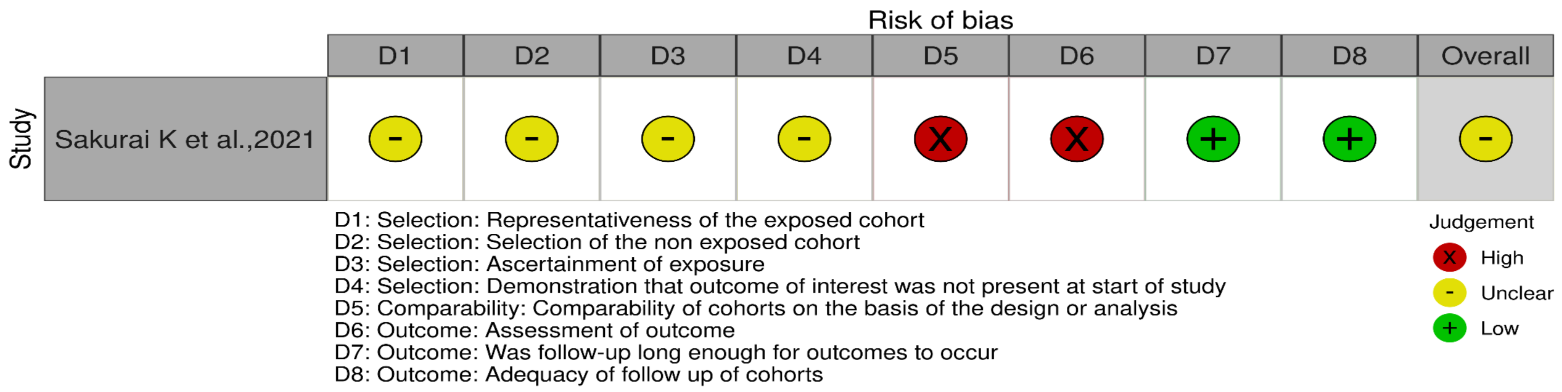

Abbreviations: ADAS-Cog, Alzheimer's Disease Assessment Scale–Cognitive Subscale; MMSE, Mini-Mental State Examination; MoCA, Montreal Cognitive Assessment; CDR, Clinical Dementia Rating; RAVLT, Rey Auditory Verbal Learning Test; WMS-DR, Wechsler Memory Scale–Delayed Recall; CDT, Clock Drawing Test; FAB, Frontal Assessment Battery; PVF, Phonemic Verbal Fluency; SVF, Semantic Verbal Fluency; BBB, blood–brain barrier; fMRI, functional magnetic resonance imaging; AD, Alzheimer's disease; MCI, mild cognitive impairment.

Certainty of evidence was assessed using the GRADE approach across five domains: risk of bias, inconsistency, indirectness, imprecision, and publication bias. Certainty levels were classified as high, moderate, low, or very low. "Some concerns" indicates potential risk of bias, while "serious" and "suspected" judgments reflect downgrading of evidence certainty based on predefined GRADE criteria.

13. Page, M.J.; McKenzie, J.E.; Bossuyt, P.M.; Boutron, I.; Hoffmann, T.C.; Mulrow, C.D.; Shamseer, L.; Tetzlaff, J.M.; Akl, E.A.; Brennan, S.E.; et al. A declaração PRISMA 2020: Diretriz atualizada para relatar revisões sistemáticas. *Rev. Panam. Salud Publica* **2022**, *46*, e112.

18. Mazza, E.; Fava, A.; Ferro, Y.; Rotundo, S.; Romeo, S.; Bosco, D.; Pujia, A.; Montalcini, T. Effect of the replacement of dietary vegetable oils with a low dose of extravirgin olive oil in the Mediterranean Diet on cognitive functions in the elderly. *J. Transl. Med.* **2018**, *16*, 10. https://doi.org/10.1186/s12967-018-1386-x.

22. Yoon, J.; Sasaki, K.; Nishimura, I.; Hashimoto, H.; Okura, T.; Isoda, H. Effects of Desert Olive Tree Pearls Containing High Hydroxytyrosol Concentrations on the Cognitive Functions of Middle-Aged and Older Adults. *Nutrients* **2023**, *15*, 3234. https://doi.org/10.3390/nu15143234.

23. Marianetti, M.; Pinna, S.; Venuti, A.; Liguri, G. Olive polyphenols and bioavailable glutathione: Promising results in patients diagnosed with mild Alzheimer's disease. *Alzheimers Dement.* **2022**, *8*, e12278. https://doi.org/10.1002/trc2.12278.

24. Kaddoumi, A.; Denney, T.S.J.; Deshpande, G.; Robinson, J.L.; Beyers, R.J.; Redden, D.T.; Praticò, D.; Kyriakides, T.C.; Lu, B.; Kirby, A.N.; et al. Extra-Virgin Olive Oil Enhances the Blood-Brain Barrier Function in Mild Cognitive Impairment: A Randomized Controlled Trial. *Nutrients* **2022**, *14*, 5102. https://doi.org/10.3390/nu14235102.

25. Tsolaki, M.; Lazarou, E.; Kozori, M.; Petridou, N.; Tabakis, I.; Lazarou, I.; Karakota, M.; Saoulidis, I.; Melliou, E.; Magiatis, P. A Randomized Clinical Trial of Greek High Phenolic Early Harvest Extra Virgin Olive Oil in Mild Cognitive Impairment: The MICOIL Pilot Study. *J. Alzheimers Dis.* **2020**, *78*, 801–817. https://doi.org/10.3233/JAD-200405.

26. Sakurai, K.; Shen, C.; Shiraishi, I.; Inamura, N.; Hisatsune, T. Consumption of Oleic Acid on the Preservation of Cognitive Functions in Japanese Elderly Individuals. *Nutrients* **2021**, *13*, 284. https://doi.org/10.3390/nu13020284.
